# Role of intracellular calcium stores in hair-cell ribbon synapse

**DOI:** 10.3389/fncel.2014.00162

**Published:** 2014-06-12

**Authors:** Manuel Castellano-Muñoz, Anthony J. Ricci

**Affiliations:** ^1^Department of Otolaryngology, Stanford University School of MedicineStanford, CA, USA; ^2^Department of Molecular and Cellular Physiology, Stanford University School of MedicineStanford, CA, USA

**Keywords:** ribbon synapse, hair cell, calcium store, CICR, hearing, synaptic plasticity

## Abstract

Intracellular calcium stores control many neuronal functions such as excitability, gene expression, synaptic plasticity, and synaptic release. Although the existence of calcium stores along with calcium-induced calcium release (CICR) has been demonstrated in conventional and ribbon synapses, functional significance and the cellular mechanisms underlying this role remains unclear. This review summarizes recent experimental evidence identifying contribution of CICR to synaptic transmission and synaptic plasticity in the CNS, retina and inner ear. In addition, the potential role of CICR in the recruitment of vesicles to releasable pools in hair-cell ribbon synapses will be specifically discussed.

Sensory transduction in the inner ear relies on the mechanoelectrical capabilities of hair cells, the sensory receptors in auditory and vestibular organs. Hair cells contain two differentiated compartments with distinct physiological roles: the mechanosensitive hair bundle and the basolateral membrane that includes specialized synaptic zones. The hair bundle is responsible for translating a mechanical vibratory input into an electrical current that, coupled with the basolateral complement of voltage and calcium activated ion channels, creates a receptor potential. The receptor potential drives synaptic output from ribbon synapses. Ribbons are vesicle-associated structures implicated in the modulation of trafficking and fusion of synaptic vesicles at presynaptic terminals (Heidelberger et al., 2002; Frank et al., 2010; Snellman et al., 2011). Ribbons are also postulated to be important for creating a large pool of primed vesicles and even perhaps recycling endosomes into reusable synaptic vesicles (Kantardzhieva et al., 2013). Calcium ions play critical but distinct roles in mechanotransduction, receptor potential modulation and synaptic transmission. Whereas Ca^2+^ defines the open probability of apical mechanotransduction channels in hair bundles (Farris et al., 2006; Johnson et al., 2011; Peng et al., 2013) and regulates basolateral membrane channels (Art and Fettiplace, 2006), Ca^2+^ levels allow the trafficking and exocytosis of neurotransmitter-containing synaptic vesicles at ribbon synapses (Moser et al., 2006). Evoked synaptic transmission is mediated by Ca^2+^ influx through voltage-dependent Ca^2+^ channels and is additionally modulated by release of Ca^2+^ from intracellular stores. In this review, we will discuss the potential contribution of intracellular stores and Ca^2+^-induced Ca^2+^ release (CICR) to synaptic transmission in central, retinal and hair-cell ribbon synapses. The potential role of CICR in the recruitment of vesicles to releasable pools in hair-cell ribbon synapses will also be addressed.

## CICR IN CENTRAL SYNAPSES

Calcium is an essential player in multiple processes in excitable cells, including the release of neurotransmitter in neurosecretory (Penner and Neher, 1988; Tse et al., 1997), central neurons (Kuba, 1994), and sensory receptors (Thoreson, 2007). During synaptic transmission, the influx of extracellular Ca^2+^ from voltage-dependent Ca^2+^ channels allows the release of neurotransmitter through the recruitment and exocytosis of vesicles in the active zone of presynaptic terminals (Katz and Miledi, 1969; Neher, 1998). However, the release of neurotransmitter can also be triggered by Ca^2+^ released from intracellular organelles containing ryanodine receptors (RyRs) or inositol triphosphate receptors (IP_3_Rs) by an amplificatory process termed CICR. In addition, recent evidences point to a role for Ca^2+^ released by endolysosomal vesicles containing NAADP-gated two-pore channels in central synaptic transmission (Chameau et al., 2001; Calcraft et al., 2009; Zhu et al., 2010), though this mechanism remains to be investigated at ribbon synapses. CICR has a role in both presynaptic (Llano et al., 2000; Unni et al., 2004) and postsynaptic terminals (Savic and Sciancalepore, 1998; Llano et al., 2000; Emptage et al., 2001; Rose and Konnerth, 2001; Simkus and Stricker, 2002). In neurons, CICR is implicated in neuronal excitability, gene expression, synaptic plasticity and synaptic release (Bouchard et al., 2003; Verkhratsky, 2005; Parekh, 2008). Endoplasmic reticulum (ER) seems to be the intracellular Ca^2+^ store responsible for CICR in presynaptic terminals (Verkhratsky, 2005). The neuronal ER is a continuous network that spreads throughout the cell, including soma, axons, boutons, dendrites and spines (Berridge, 1998; Chen et al., 2014). Although not entirely conclusive, there are anatomical evidences for the presence of ER along with IP_3_Rs and RyRs in presynaptic terminals (Ungar et al., 1981; Mercurio and Holtzman, 1982; Ungar et al., 1984; Krijnse-Locker et al., 1995; Bouchard et al., 2003). Neuronal RyRs can be activated following Ca^2+^ influx through voltage-dependent Ca^2+^ channels or ionotropic glutamate receptors (Berridge, 1998). Despite the lack of a unified mechanistic model, presynaptic CICR is present in several neuronal types such as motor neurons (Erulkar and Rahamimoff, 1978; Soga-Sakakibara et al., 2010), sympathetic neurons (Hua et al., 1993), cerebellar basket cells (Galante and Marty, 2003), striatal neurons (Plotkin et al., 2013), thalamocortical neurons (Cheong et al., 2011), Purkinje cells (Llano et al., 2000), sensory neurons (Shmigol et al., 1995), and cochlear nucleus neurons (Kato and Rubel, 1999). The presence of action potential-evoked as well as spontaneous CICR has been demonstrated by pharmacological effects of ryanodine, caffeine and other drugs on presynaptic Ca^2+^ levels and postsynaptic currents. At presynaptic terminals, Ca^2+^ stores modulated action potential-evoked Ca^2+^ signals, regulating the efficacy of transmitter release (Galante and Marty, 2003; Collin et al., 2005). In hippocampal boutons, action potentials evoked large Ca^2+^ transients triggered by both influx from Ca^2+^ channels and Ca^2+^ released from internal stores (Emptage et al., 2001). The observation that the frequency of spontaneous miniature postsynaptic events (mEPSCs) was reduced when blocking CICR in cortical (Simkus and Stricker, 2002), hippocampal (Emptage et al., 2001), and cerebellar neurons (Llano et al., 2000; Bardo et al., 2002), suggested that spontaneous exocytosis requires Ca^2+^ release from internal stores (Emptage et al., 2001). In motor neuron terminals, CICR was primed by tetanic stimulation, increasing the frequency of spontaneous release events (Narita et al., 1998). In cerebellar interneuron–Purkinje cell synapses, spontaneous presynaptic Ca^2+^ transients, reminiscent of Ca^2+^ sparks in muscle, were reduced by ryanodine (Llano et al., 2000). Interestingly, large-amplitude miniature inhibitory postsynaptic currents (mIPSCs; maximinis) persisted in the presence of tetrodoxin, cadmium or Ca^2+^-channel toxins, suggesting the contribution of CICR to spontaneous presynaptic activity (Llano et al., 2000). In reciprocal synapses between retinal amacrine cells and rod bipolar cells, Ca^2+^ influx through amacrine cell AMPARs triggers the synaptic release of GABA through CICR (Chavez et al., 2006).

Unfortunately, the main caveat in the identification of the physiological role of CICR in synaptic transmission is that most experimental evidences exclusively rely on the effects of non-specific pharmacological agents. In addition, these drugs have often opposite effects depending on the dose and exert both pre and post-synaptic effects, adding complexity to pharmacological results that lead to ambiguous conclusions (Barnes and Hille, 1989; Llano et al., 2000; Emptage et al., 2001; Rose and Konnerth, 2001; Bouchard et al., 2003; Collin et al., 2005; Bardo et al., 2006). In fact, different reports using similar pharmacological approaches have resulted in contradictory conclusions (Emptage et al., 2001; Carter et al., 2002; Lelli et al., 2003; Beurg et al., 2005; Cadetti et al., 2006; Suryanarayanan and Slaughter, 2006). An additional source of variability in the study of CICR relies on the existence of different isoforms of RyRs (Lanner et al., 2010). Furthermore, drugs used in the study of CICR can also alter other Ca^2+^ homeostatic mechanisms, such as store-operated Ca^2+^ entry (SOCE), which can also modulate neuronal (Emptage et al., 2001) and ribbon synaptic transmission (Szikra et al., 2008). Nevertheless, numerous evidences point to the existence of a bona fide physiological role for CICR in synaptic transmission, although the distinct mechanism remains unclear (Hua et al., 1993; Llano et al., 2000; Sharma and Vijayaraghavan, 2003; Collin et al., 2005; Gordon and Bains, 2005; Bardo et al., 2006).

## ROLE OF CICR IN CENTRAL SYNAPTIC TRANSMISSION

In neurons and cardiac cells, CICR is a graded rather than an all-or-none phenomenon in which release of Ca^2+^ from stores increases in a graded fashion with increasing stimulus strength (Ca^2+^ channel activation; Fabiato, 1985; Beuckelmann and Wier, 1988; Hua et al., 1993; Usachev and Thayer, 1997; Berridge, 1998). Additionally, neuronal regenerative CICR can also be observed by incubating with a sensitizing agent such as caffeine and applying suprathreshold electrical stimulation (Usachev and Thayer, 1997). Local Ca^2+^ nanodomains generated by activation of close Ca^2+^ channels are sufficient to induce Ca^2+^ release from the ER (Stern, 1992; Stern et al., 1999). Ca^2+^ influx and efflux microdomains may exist as separate identities in the ER. Presynaptically, such spatial distribution suggests that different active zones could activate RyRs independently to coordinate intracellular release through the lumen of the ER network (Friedman and Voeltz, 2011; Chen et al., 2014). This scenario is consistent with multiple coincident exocytic events triggered by Ca^2+^ stores instead of nearby Ca^2+^ channels. Multivesicular release and compound fusion of synaptic vesicles is reported in central neurons (Tong and Jahr, 1994; He et al., 2009). Presynaptic information can be linearly transmitted to the postsynaptic terminal through multivesicular release in those synapses where receptors are not saturated nor desensitized (Singer et al., 2004), thus supporting short-term synaptic plasticity (Oertner et al., 2002; Quinlan and Hirasawa, 2013). Moreover, multivesicular release caused large miniature postsynaptic currents (*maximinis*) as large as 1 nA in cerebellar Purkinje cells (Llano et al., 2000). Ryanodine (100 μM) decreased the amplitude and frequency of mIPSCs and selectively eliminated *maximinis*, indicating that presynaptic RyRs are involved in the generation of multivesicular release (Llano et al., 2000). Extracellular Ca^2+^ removal abolished the presence of these *maximinis* and reduced the mean amplitude of uniquantal mIPSCs, suggesting that presynaptic Ca^2+^ reduction leads to depletion of Ca^2+^ stores and disruption of multivesicular release (Llano et al., 2000).

Together with cytoplasmic Ca^2+^ buffering, extrusion through plasma membrane ATPases and uptake into organelles, release of Ca^2+^ from intracellular stores contributes to the control of cytoplasmic basal Ca^2+^ levels. However, ER Ca^2+^ levels further confer *memory* of previous activity (Berridge, 1998). The amount of releasable Ca^2+^ from neuronal ER is proportional to the Ca^2+^ load contained in its lumen, which in turn depends on the cytosolic Ca^2+^ levels (Sitsapesan and Williams, 1990; Hua et al., 1993; Garaschuk et al., 1997; Berridge, 1998; Krizaj et al., 1999). Furthermore, RyR sensitivity is augmented by high luminal Ca^2+^ levels (Fill and Copello, 2002). Therefore, the amount of Ca^2+^ sequestered in the ER may become larger after consecutive stimulation and dependent on the magnitude of prior release. Intracellular Ca^2+^ stores have a role in neuronal synaptic plasticity (Zucker, 1989). At neuromuscular junctions, inhibitors of mitochondrial Ca^2+^ uptake and release blocked post-tetanic potentiation (Tang and Zucker, 1997). Nevertheless, numerous experimental results suggests that the ER is a highly dynamic intracellular Ca^2+^ store ideally suited for regulating different forms of synaptic plasticity (Fitzjohn and Collingridge, 2002; Baker et al., 2013). In addition, CICR has been implicated in long-term forms of synaptic plasticity. Genetic or pharmacological disruption of RyRs enhanced long-term potentiation (LTP) and impaired long-term depression (LTD) in CA1 pyramidal neurons [Futatsugi et al., 1999; Nishiyama et al., 2000; but also see Qin et al. (2012)], suggesting that Ca^2+^ from intracellular stores may contribute to decrease the threshold for LTD expression (Reyes and Stanton, 1996). Since sustained moderate Ca^2+^ rise induces NMDAR-dependent depression of synaptic transmission, postsynaptic steady-state CICR might support LTD under continuous synaptic activity, maybe through the recruitment of a subpopulation of AMPAR-containing vesicles. According to this, NMDAR-dependent LTD required Ca^2+^ release from ryanodine-sensitive stores in CA3–CA3 hippocampal synapses (Unni et al., 2004). Furthermore, the coupling of postsynaptic AMPARs and Ca^2+^ stores could adjust synaptic strength depending on the number of synchronically activated synapses, switching the direction of synaptic plasticity from LTP to LTD (Camire and Topolnik, 2014). Similarly, both pre and postsynaptic ryanodine-sensitive Ca^2+^ stores were necessary for LTD induction in hippocampal GABAergic synapses (Caillard et al., 2000). LTP is reported to rely on IP_3_Rs in presynaptic ER of sympathetic ganglia synapses (Cong et al., 2004). Moreover, synaptic plasticity can be modulated by the control of Ca^2+^-dependent vesicle mobilization between different vesicle pools (Alabi and Tsien, 2012), a mechanism potentially regulated by presynaptic Ca^2+^ stores (Levitan, 2008).

Most synapses with low probability of release show synaptic facilitation, a short-term form of plasticity in which repeated stimulation leads to a transient increase in the probability of synaptic release. CICR can sustain paired-pulse facilitation (PPF), a form of plasticity lasting hundreds of milliseconds to seconds. During PPF, the amplitude of a second excitatory postsynaptic potential (EPSP) becomes larger than a first EPSP, a phenomenon often attributed to residual Ca^2+^ from the first pulse summing up with the second pulse (Katz and Miledi, 1968). In pyramidal hippocampal neurons, blocking CICR with ryanodine or cyclopiazonic acid (CPA) reduced the enhancement in the second stimulus when separated by tens of milliseconds (Emptage et al., 2001) suggesting that CICR may be the source of some residual calcium. Thapsigargin also suppressed synaptic facilitation during high-frequency stimulation in hippocampal synapses (Zhang et al., 2009), further pointing to a role of CICR in short-term plasticity. The recruitment of reserve vesicles near active zones could be the functional target of CICR, modulating synaptic strength and mediating certain forms of synaptic plasticity (Bardo et al., 2006). However, mobilization of vesicles from the reserve pool has also been attributed to Ca^2+^ leakage from mitochondrial stores (Billups and Forsythe, 2002; Storozhuk et al., 2005), suggesting that short-term vesicle mobilization can be modulated by multiple Ca^2+^ stores. Nevertheless, the role of CICR in short-term synaptic plasticity remains controversial (Carter et al., 2002), and more data are needed to clarify the role of CICR on the milliseconds-to-seconds time scale.

## CICR IN PHOTORECEPTOR RIBBON SYNAPSES

Aside from central synapses, CICR also plays a role in ribbon synaptic transmission in the retina and inner ear. Ribbon synapses support fast and sustained transmission of graded inputs through multivesicular release of synaptic vesicles in unique presynaptic organelles called synaptic ribbons. The existence of CICR was demonstrated in rods and cones, the two types of photoreceptors (Krizaj et al., 1999; Suryanarayanan and Slaughter, 2006; Szikra and Krizaj, 2006). In rods, a depolarization-evoked intracellular Ca^2+^ rise spread from the active zone across the synaptic terminal and could be blocked by ryanodine, suggesting that CICR has a presynaptic role in retinal synaptic transmission (Cadetti et al., 2006). Blocking Ca^2+^ sequestration into the ER by thapsigargin and CPA, two sarco/ER Ca^2+^ ATPase (SERCA) inhibitors, decreased the magnitude of depolarization-evoked and caffeine-evoked presynaptic Ca^2+^ transients, pointing to the ER as the intracellular Ca^2+^ store involved in CICR at photoreceptors (Szikra and Krizaj, 2007). Although its physiological effect varies among preparations, ryanodine generally promotes RyR opening at low micromolar concentrations (around 1–5 μM) whereas it blocks RyRs at higher concentrations (50–100 μM; Verkhratsky, 2005). Ryanodine (10 μM) increased cytoplasmic Ca^2+^ levels in somas and synaptic terminals of rods, consistent with its effect as RyR agonist (Babai et al., 2010b). Similarly, caffeine, which is known to sensitize RyRs to Ca^2+^, triggered a robust transient Ca^2+^ increase followed by prolonged reduction (Krizaj et al., 1999). Ryanodine (20 μM) suppressed these caffeine-evoked effects (Krizaj et al., 1999). In addition, Ca^2+^ substitution by barium, a divalent ion which is poorly sequestered into stores (Kwan and Putney, 1990; Adachi-Akahane et al., 1996), also suppressed caffeine effects (Krizaj et al., 1999). Immunohistochemistry studies showed RyRs in terminals of cones and rods (Krizaj et al., 2003, 2004), along with partial colocalization between ER-containing Ca^2+^ ATPase SERCA2 and Ribeye, the most abundant protein in synaptic ribbons (Babai et al., 2010b). All these data support the existence of CICR in photoreceptors and point to a real function of Ca^2+^ stores in phototransduction. In addition to CICR, SOCE also contribute to ribbon synaptic transmission by regulating presynaptic Ca^2+^ homeostasis (Szikra et al., 2008, 2009).

Evidence for the role of presynaptic CICR in ribbon synaptic transmission was obtained at rod-horizontal cell (Cadetti et al., 2006) and rod-bipolar cell ribbon synapses (Suryanarayanan and Slaughter, 2006). Mitochondrial Ca^2+^ uptake occurs in photoreceptors and bipolar presynaptic terminals only after strong depolarization (Zenisek and Matthews, 2000; Krizaj et al., 2003; Wan et al., 2012). However, the major role of mitochondria near synaptic ribbons is to provide the energy needed to pump Ca^2+^ outside the terminal (Zenisek and Matthews, 2000; Linton et al., 2010). Unlike mitochondria, the ER is directly involved in presynaptic CICR at retinal ribbon synapses. Caffeine incubation elicited a brief depolarization followed by a progressive hyperpolarization in horizontal cells, suggesting that presynaptic caffeine-sensitive Ca^2+^ stores modulate neurotransmitter release at the ribbon synapse (Krizaj et al., 1999). Similarly, light-evoked currents in horizontal cells were reduced by ryanodine (100 μM), demonstrating the importance of CICR in this synapse and suggesting a potential presynaptic site of action (Babai et al., 2010b). In paired recordings from rods, blocking CICR with ryanodine reduced the later portions of the horizontal cell EPSC (Cadetti et al., 2006; Suryanarayanan and Slaughter, 2006). This effect was more prominent after prolonged stimulation, pointing to a key role of CICR on the later components of synaptic release (Cadetti et al., 2006). Ryanodine also reduced the intraretinal b-wave, the bipolar cell-evoked component of electroretinogram (Babai et al., 2010b). Light-evoked postsynaptic currents in bipolar cells were abolished by 100 μM ryanodine (Suryanarayanan and Slaughter, 2006). In rod-bipolar synapses, ryanodine reduced both phasic and sustained transmitter release at rod physiological potentials (Suryanarayanan and Slaughter, 2006). The predominant effect of ryanodine on the late part of the bipolar EPSCs at different rod potentials pointed to a direct physiological role of CICR in sustained exocytosis of synaptic vesicles at rod terminals. Moreover, caffeine addition while blocking presynaptic Ca^2+^ channels with cadmium, evoked EPSCs in bipolar cells (Suryanarayanan and Slaughter, 2006), suggesting that Ca^2+^ released from stores is capable of evoking EPSCs. In this view, continuous moderate activation of voltage-dependent calcium channels would activate CICR-dependent exocytosis of vesicles, whereas strong stimulation could trigger vesicle exocytosis directly due to abrupt larger calcium loads through voltage-dependent calcium channels. This hypothesis points to a role for CICR in the resupply of vesicles for exocytosis during continuous stimulation. The large spontaneous postsynaptic events observed in paired recordings likely represents coordinated release of multiple vesicles (Suryanarayanan and Slaughter, 2006). The frequency and amplitude of these spontaneous EPSCs was reduced by ryanodine, suggesting a role for CICR in coordinating multivesicular release (Suryanarayanan and Slaughter, 2006). Bipolar cells present two EPSC components in response to rod depolarization: a transient and a sustained component of slower onset. The sustained synaptic component was reduced by 100 μM ryanodine or 5 mM BAPTA, pointing to a role for CICR in the exocytosis of vesicles recruited from a reserve pool (Suryanarayanan and Slaughter, 2006). Addition of ryanodine (100 μM) also reduced the size of the larger spontaneous EPSCs, thought to emerge from coordinated neurotransmitter release, suggesting the potential role of CICR in synchronizing the fusion of multiple vesicles (Suryanarayanan and Slaughter, 2006). Since the recruitment of vesicles involves sites located a few hundreds of nanometers from the Ca^2+^ channels, it is possible that vesicle trafficking is modulated by the summation of Ca^2+^ signals from multiple nanodomains. This would reduce noise from stochastic Ca^2+^ channel openings, improving the signal-to-noise ratio and allowing CICR to ultimately regulate sustained release. Alternatively, CICR could facilitate the coordinated fusion of vesicles far from the active zone. Extrasynaptic exocytosis of vesicles or prefusion of vesicles before reaching the active zone are alternative scenarios consistent with this idea. Supporting this view, long depolarization pulses (>200 ms) applied to rods evoked the exocytosis of vesicles located far from synaptic ribbons (Chen et al., 2013). These ectopic exocytic events were triggered by CICR, suggesting the potential contribution of ER to maintained synaptic release of vesicles (Chen et al., 2014). By contrast, ectopic ribbon-independent synaptic release in rod bipolar-AII amacrine cell synapses was independent on intracellular calcium stores (Mehta et al., 2014), suggesting differential CICR contributions to different types of ribbon synapses.

## CICR IN HAIR CELLS

The release of Ca^2+^ from intracellular stores has been demonstrated in hair cells of different animals in auditory and vestibular organs, where pharmacological modulators of Ca^2+^ stores and RyRs exerted an effect on Ca^2+^ hotspot amplitude, Ca^2+^ basal levels, membrane ion channels or hair-cell membrane capacitance. Imaging and electrophysiological experiments showed the existence of CICR in hair cells of frog semicircular canal (Hendricson and Guth, 2002; Lelli et al., 2003), turtle auditory papilla (Tucker and Fettiplace, 1995; Schnee et al., 2011b), and mammalian inner hair cells (IHCs; Kennedy and Meech, 2002) and outer hair cells (OHCs; Lioudyno et al., 2004).

In vestibular hair cells of the frog semicircular canal, caffeine (10 mM) increased intracellular Ca^2+^ levels, an effect that was diminished by ryanodine (40 μM; Lelli et al., 2003). Additionally, incubation with caffeine (1 mM) potentiated depolarization-evoked Ca^2+^ transients in hair-cell hotspots of the semicircular canal. Conversely, ryanodine (40 μM) reduced depolarization-evoked Ca^2+^ transients. In a minority of cells, caffeine (500 μM) also evoked membrane capacitance increases whereas ryanodine (40 μM) reduced voltage-dependent capacitance increases (Lelli et al., 2003). Interestingly, this reduction was more apparent after repeated stimulation. The presence of RyRs in vestibular hair cells was also suggested by immunohistochemical evidence (Perin et al., 2012). In addition, the contribution of CICR to hair-cell synaptic transmission was further confirmed at postsynaptic compartments. The dose-dependent reduction of semicircular canal afferent discharge after incubation of caffeine, ryanodine and thapsigargin suggested a physiological role for Ca^2+^ stores in vestibular synaptic transmission (Hendricson and Guth, 2002). Postsynaptically, caffeine (20 mM) increased and ryanodine (1 mM) decreased spontaneous action potentials in the vestibular nerve (Lelli et al., 2003). The complete reduction of multiunit vestibular afferent discharge after incubation of xestospongin C (1 μM), an IP_3_R blocker, also pointed to the presence of pre or postsynaptic IP_3_-sensitive Ca^2+^ stores (Hendricson and Guth, 2002). Similarly, IP_3_R inhibitors and compounds that increase IP_3_ production modulated vestibular discharge in frog semicircular canal (Rossi et al., 2006).

**FIGURE 1:**
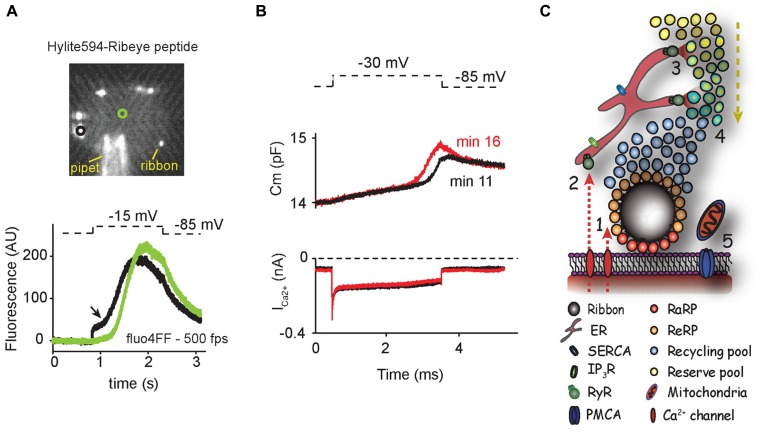
**(A)** Hair cells show non-linear calcium increases during depolarization. Upper panel shows a swept-field confocal image of a single turtle auditory hair cell during patch clamp experiments. Hylite594-conjugated ribbon-binding peptide in the internal solution defined the location of synaptic ribbons for subsequent calcium imaging recordings (note that peptide also binds unspecifically to the pipet). In the lower panel, calcium levels were monitored in response to depolarization by swept-field confocal microscopy at 500 frames per second (fps) using the low-affinity calcium dye fluo4FF. Black and green traces correspond to intracellular locations near and far from synaptic ribbons (circles in upper panel). Arrow points the onset of supralinear calcium rise. **(B)** Hair cells show time-variant exocytic enhancement. In an independent experiment, calcium currents and real-time membrane capacitance were obtained by dual sine capacitance methods. Eleven minutes after whole cell configuration, a superlinear release was first obtained during prolonged moderate depolarization (black trace). The onset of superlinear release was shifted by equivalent calcium load 5 min later (red trace). **(C)** Intracellular calcium stores could modulate the recruitment of vesicles to the plasma membrane. Calcium influx through L-type calcium channels triggers exocytosis of synaptic vesicles near the active zone (1). In parallel, calcium also activates RyRs (2), triggering the release of calcium stored in the ER at sites far from the ribbon (3). CICR allows the recruitment of vesicles from reserve pools to the vicinity of the ribbon (4). (ER = endoplasmic reticulum; IP_3_R = inositol triphosphate receptor; SERCA = sarco/endoplasmic reticulum calcium ATPase; RaRP = rapid releasable pool; ReRP = readily releasable pool.) Mitochondria and calcium pumps maintain homeostatic calcium levels in cytoplasm (5). Arrows depict the direction of calcium influx and vesicle trafficking.

Hair cells of the basilar papilla, the auditory organ of reptiles, amphibians and birds also contain intracellular Ca^2+^ stores. BHQ (50 μM), a specific blocker of ER CaATPase, and caffeine (20 mM) elevated Ca^2+^ levels throughout the cell (Tucker and Fettiplace, 1995; Tucker et al., 1996), confirming the presence of ER Ca^2+^ stores. BHQ and thapsigargin prolonged the duration of Ca^2+^-dependent SK currents (Tucker et al., 1996) and increased Ca^2+^-dependent inactivation of L-type Ca^2+^ channels (Lee et al., 2007). These results confirm that ER Ca^2+^ homeostasis has an influence near the hair-cell membrane and suggest that the Ca^2+^ buffering and releasing capabilities of the ER might be separated spatially to avoid functional interference. More recently, a non-linear rise in the intracellular Ca^2+^ levels obtained by high-speed Ca^2+^ imaging during prolonged depolarization further pointed to the existence of CICR (Schnee et al., 2011b; **Figure 1A**).

Intracellular Ca^2+^ stores were identified in IHCs of the mammalian cochlea. Inhibitors of ER Ca^2+^ uptake such as BHQ (100 μM), thapsigargin (200 nM), or CPA (30 μM) increased resting cytoplasmic Ca^2+^ levels and slowed the recovery time after brief depolarization in neonatal IHCs (Kennedy, 2002). An increase in intracellular Ca^2+^ baseline levels was also observed after 10 mM caffeine application in adult guinea pigs IHCs (Beurg et al., 2005), but not after 5 mM caffeine application in immature mice IHCs (Kennedy, 2002). Ryanodine (20 μM) reduced both amplitude and rise rate of depolarization-evoked Ca^2+^ transients in neonatal IHCs (Kennedy and Meech, 2002), a result that could not be reproduced in IHCs from 14 to 18 days-old mice (Frank et al., 2009). The amplitude of Ca^2+^-dependent K^+^ currents was reduced by ryanodine (20–100 μM) or caffeine and thapsigargin (1 μM), demonstrating that the activity of RyRs located in the ER modulate Ca^2+^ levels around plasma membrane channels in mature IHCs (Marcotti et al., 2004; Beurg et al., 2005). In fact, immungold-EM localized RyRs in the ER at the base of IHCs and cisternae of the basolateral membrane (Grant et al., 2006). Compound action potentials in the auditory nerve were reversibly inhibited by intracochlear perfusion of ryanodine (50–100 μM), further confirming the physiological relevance of hair-cell CICR in synaptic transmission (Beurg et al., 2005). Substitution of K^+^ by cesium (Cs^+^) in the intracellular pipette solution is often used in patch-clamp experiments to block K^+^ conductances and isolate Ca^2+^ currents. Interestingly, Cs^+^ reduced Ca^2+^ increase in IHCs by an unknown intracellular mechanism, apparently blocking CICR (Kennedy and Meech, 2002). However, this effect was not observed in basilar papilla hair cells (Schnee et al., 2011b).

Mammalian OHCs present a robust CICR mechanism associated to efferent innervation (Ashmore and Ohmori, 1990; Sridhar et al., 1997; Evans et al., 2000; Lioudyno et al., 2004; Grant et al., 2006). Similarly, application of ATP to the hair bundle of OHCs triggered the release of Ca^2+^ from IP_3_R-containing apical intracellular stores (Mammano et al., 1999). In addition to their electromotile behavior that supports cochlear amplification of incoming sounds, OHCs support synaptic transmission to type II afferents in response to loud sounds (Brown, 1994; Weisz et al., 2012). A potential role for CICR in type II afferents synaptic transmission has never been experimentally addressed.

## ROLE OF CICR IN HAIR-CELL SYNAPTIC TRANSMISSION

Despite the numerous reports demonstrating the presence of intracellular Ca^2+^ stores in hair cells, their precise physiological role has not been clarified (Tucker and Fettiplace, 1995; Evans et al., 2000; Hendricson and Guth, 2002; Kennedy, 2002; Lelli et al., 2003; Marcotti et al., 2004; Beurg et al., 2005). The observation that Ca^2+^ stores regulated extrasynaptic BK channels lead to the hypothesis that CICR could counteract elevated Ca^2+^ accumulation through BK channel activation to hamper synaptic transmission during sound overstimulation or ischemia (Beurg et al., 2005). Several lines of evidence, however, lead to the proposition that Ca^2+^ stores are involved in hair-cell ribbon synaptic transmission. Synaptic transmission in auditory hair cells is characterized by an unusual broad distribution in the size of postsynaptic EPSCs, a unique feature that likely originates from the synchronized fusion of synaptic vesicles. The large range of vesicle sizes observed could additionally be due to prefusion of synaptic vesicles (Schnee et al., 2013). In turtle hair cells, lowering external Ca^2+^ dramatically reduced the frequency and size of burst-like EPSCs (Schnee et al., 2013), but the potential contribution of Ca^2+^ released from intracellular stores to complex EPSCs remains to be experimentally tested. The open probability of voltage-dependent Ca^2+^ channels controls the transient changes in presynaptic Ca^2+^ levels, allowing fast conduction of transient auditory information in adult animals (Brandt et al., 2005; Fedchyshyn and Wang, 2005). Calcium stores set the open probability of L-type Ca^2+^ channels, potentially modulating spontaneous release from ribbon synapses (Lee et al., 2007). Inhibition of ER Ca^2+^ pumps with BHQ or thapsigargin lead to faster and increased Ca^2+^ channel inactivation (Lee et al., 2007). Unlike Ca^2+^ channels in mammalian IHCs, Ca^2+^ channels in bird and turtle auditory hair cells were half inactivated at resting potentials (Schnee and Ricci, 2003; Lee et al., 2007), opening the possibility of a presynaptic control of synaptic plasticity through the regulation of Ca^2+^ channel inactivation by calcium stores. Despite the lack of evidence that Ca^2+^ stores are indeed located in close proximity to active zones, Ca^2+^ stores could modulate the Ca^2+^ concentration sensed near plasma membrane channels (Marcotti et al., 2004; Beurg et al., 2005). Therefore, although a physiological role for CICR in tonic synaptic transmission would appear to be more feasible, a contribution to phasic transmission cannot be ruled out.

Although the existence of CICR in hair cells has been ratified in different studies, a unifying theory that enlightens its physiological relevance in synaptic transmission is still missing. The recent development of new technologies is opening new avenues for the understanding of the role of CICR in synaptic transmission at hair-cell ribbon synapses (Schnee et al., 2011a; Castellano-Munoz et al., 2012). Dual sine capacitance experiments, which monitor vesicle fusion in real time, identified two different release components during prolonged depolarization (Schnee et al., 2011b). A first linear component, proportional to the Ca^2+^ load, was equivalent in size to the fusion of the pool of vesicles near the synaptic ribbon (**Figure 1B**). The onset of a later larger superlinear component was Ca^2+^-load dependent and resembled non-linear exocytic components reported in bipolar and chromaffin cells (Seward et al., 1996; Wan et al., 2010). This superlinear component could represent local buffer saturation leading to endosomal exocytosis (Coggins et al., 2007). Alternatively, this superlinear component could represent the fusion of newly recruited vesicles from the reserve pool evoked by CICR. In this scenario, reserve vesicles would be recruited by the release of stored Ca^2+^ at a distance of the active zones (**Figure 1C**). The two-kinetics behavior observed in capacitance recordings was paralleled by high-speed Ca^2+^ imaging experiments, where a supralinear intracellular Ca^2+^ rise was initiated at the vicinity of the ribbon and subsequently spread throughout the hair-cell cytoplasm (**Figure 1A**; Schnee et al., 2011b). In this view, it is conceivable that the exocytosis rate of vesicles from the readily releasable pool (RRP) with the terminal could be dependent on the Ca^2+^ load carried by voltage-dependent Ca^2+^ channels whereas the recruitment onset of reserve vesicles would be dependent on the onset of intracellular Ca^2+^ release (in turn triggered by plasma membrane Ca^2+^ influx). These two mechanisms would work in parallel to allow both phasic and tonic transmitter release for prolonged periods of time. In fact, the reduction in Ca^2+^ transients and exocytosis in hair cells by ryanodine application was more apparent after repeated stimulation, pointing to a CICR effect on the reserve pool of vesicles (Lelli et al., 2003). According to this idea, the existence of a Ca^2+^-dependent mechanism necessary to speed up the supply of new vesicles to the RRP during repetitive stimulation was proposed in neurosecretory cells (von Ruden and Neher, 1993; Voets et al., 1999). Central and ribbon synapses also contain calcium-dependent mechanisms of vesicle replenishment (Dittman and Regehr, 1998; Stevens and Wesseling, 1998; Wang and Kaczmarek, 1998; Gomis et al., 1999; Shakiryanova et al., 2005) that play a direct role in encoding receptor potential into changes of sustained release rates (Jackman et al., 2009; Babai et al., 2010a). Moreover, the rate at which vesicles are recruited to the RRP depends on the levels of free Ca^2+^ and is modulated by synaptic activity (Stevens and Wesseling, 1998). These observations are consistent with a graded form of CICR being continuously triggered by Ca^2+^ influx, allowing the resupply of synaptic vesicles to active zones. Temporal regulation of vesicle recruitment may uncover new forms of presynaptic plasticity in hair-cell ribbon synapses (**Figure 1B**; Alabi and Tsien, 2012). It is important to note that the experimental dissection of the kinetics of both fusion and recruitment of reserve synaptic vesicles is only possible by the use of hyperpolarizing holding potentials used in patch-clamp experiments (Schnee et al., 2011b). Unlike most central neurons, these potentials do not represent physiological resting conditions, since hair cells are moderately depolarized at rest due to the contribution of the mechanotransduction channel in the hair bundle. Conversely, more physiological holding potentials showed an overlapping of both components (Schnee et al., 2011b). Moreover, prepulse experiments using depolarization steps preceded by physiological resting potentials demonstrated an increase in exocytosis along with an increase in synaptic strength and a reduction in synaptic latency (Goutman and Glowatzki, 2011; Schnee et al., 2011b). All these experiments suggest that incessant Ca^2+^ influx allows hair-cell ribbon synapses to operate in a continuously facilitated mode at resting membrane potentials, thus optimizing the timing and size of postsynaptic responses in the auditory nerve. Future experiments are needed to address the nature and physiological relevance of both the exocytic superlinear component and the non-linear intracellular Ca^2+^ rises in the recruitment of vesicles mediated by CICR for hair-cell ribbon synaptic transmission.

## Conflict of Interest Statement

The authors declare that the research was conducted in the absence of any commercial or financial relationships that could be construed as a potential conflict of interest.
